# Neurophysiological evidence that frontoparietal connectivity and GABA-A receptor changes underpin the antidepressant response to ketamine

**DOI:** 10.1038/s41398-024-02738-w

**Published:** 2024-02-24

**Authors:** Rachael L. Sumner, Rebecca L. McMillan, Anna Forsyth, Suresh D. Muthukumaraswamy, Alexander D. Shaw

**Affiliations:** 1 School of Pharmacy, University of Auckland, Auckland, New Zealand; 2 School of Psychology, University of Exeter, Exeter, UK

**Keywords:** Depression, Neuroscience

## Abstract

Revealing the acute cortical pharmacodynamics of an antidepressant dose of ketamine in humans with depression is key to determining the specific mechanism(s) of action for alleviating symptoms. While the downstream effects are characterised by increases in plasticity and reductions in depressive symptoms—it is the acute response in the brain that triggers this cascade of events. Computational modelling of cortical interlaminar and cortico-cortical connectivity and receptor dynamics provide the opportunity to interrogate this question using human electroencephalography (EEG) data recorded during a ketamine infusion. Here, resting-state EEG was recorded in a group of 30 patients with major depressive disorder (MDD) at baseline and during a 0.44 mg/kg ketamine dose comprising a bolus and infusion. Fronto-parietal connectivity was assessed using dynamic causal modelling to fit a thalamocortical model to hierarchically connected nodes in the medial prefrontal cortex and superior parietal lobule. We found a significant increase in parietal-to-frontal AMPA-mediated connectivity and a significant decrease in the frontal GABA time constant. Both parameter changes were correlated across participants with the antidepressant response to ketamine. Changes to the NMDA receptor time constant and inhibitory intraneuronal input into superficial pyramidal cells did not survive correction for multiple comparisons and were not correlated with the antidepressant response. These results provide evidence that the antidepressant effects of ketamine may be mediated by acute fronto-parietal connectivity and GABA receptor dynamics. Furthermore, it supports the large body of literature suggesting the acute mechanism underlying ketamine’s antidepressant properties is related to GABA-A and AMPA receptors rather than NMDA receptor antagonism.

## Introduction

It has been argued that the discovery of the rapidly acting antidepressant action of ketamine is the most important development in depression research in the last 50 years [1]. The first empirical evidence was provided by Berman and Cappiello [2] who demonstrated the efficacy of a single, subanaesthetic intravenous (IV) infusion of ketamine (0.5 mg/kg) over 40 min in treating major depressive disorder (MDD). Ketamine’s antidepressant properties have now been replicated many times and it has been empirically shown ketamine demonstrates efficacy across patients, including those deemed treatment-resistant to conventional antidepressants [3]. Identifying the acute pharmacodynamic effect of ketamine that precedes its subsequent antidepressant effects has remained an open research question.

Ketamine is often described simply as an N-methyl-d-aspartate (NMDA) receptor antagonist, however, the failure of other NDMA antagonists such as memantine [4] to exert antidepressant benefits indicate that the complex and possibly synergistic pharmacological effects of ketamine are required to elicit an antidepressant response. Candidate direct effects include ketamine preferentially binding to the NMDA receptors that densely populate γ-aminobutyric acid (GABA)-mediated interneurons [5]. Candidate indirect effects include the consequent disinhibition of α-amino-3-hydroxy-5-methyl-4-isoxazolepropionic acid (AMPA) receptors on the post-synaptic neuron with flow-on upregulation of excitation and the triggering of pro-plasticity cascades [5].

These pharmacodynamic effects of ketamine are likely to interact at the meso- and macroscale via changing cortical and cortico-cortical connectivity. MDD is often described as a disorder characterised by abnormal connectivity of the brain’s resting state networks [6, 7], and brain network hypotheses of depression suggest that antidepressant treatments may exert therapeutic benefits through the return of resting-state network connectivity to optimal levels [8, 9]. While it has been posited that ketamine’s acute effects on frontoparietal connectivity may play a role in its antidepressant properties, this has been inferred from healthy samples and has never been tested in patients with MDD [10–13].

Here, we recorded resting-state electroencephalography (EEG) during a subanaesthetic, antidepressant infusion of ketamine in patients with MDD. We set out to determine the acute changes to cortical microcircuitry and GABA, AMPA and NMDA receptors that generate the spectral effects on the EEG using generative computational modelling. To date modelling has only been used to assess the long-term (3–9 h post-infusion) effects in people with depression [14–17], or effects in people without depression [10, 18]. The model used here was more complex [18], more thoroughly parameterising thalamic reciprocal connectivity with the cortex, as well as separate superficial and deep interneuronal input. GABA-A, AMPA and NMDA receptors are modelled as in Moran, Symmonds [19]. Additionally, we investigated whether the acute disruption of frontoparietal connectivity by ketamine found in healthy participants [10–13] is also found in patients with MDD. Further, whether connectivity changes in the acute phase of ketamine infusion are related to antidepressant response. Lastly, a dataset of healthy young males [13] who underwent an identical EEG recording and ketamine infusion protocol is also modelled for comparison.

Successfully modelling activity of the acute effects of ketamine that are thought to mediate its antidepressant response will not only support and provide human translation of pre-clinical work surmising the antidepressant properties of ketamine, but also provide a benchmark upon which to validate the pharmacodynamic and therapeutic effects of novel drugs developed from, or informed by ketamine. Such techniques could be used to monitor the predicted effect in the brain and developed alongside pharmacokinetic modelling to support dose finding.

## Methods

### Participants

The main dataset used is from a study that was a randomised, double-blind, active placebo-controlled crossover design in 30 participants who met DSM-IV criteria for major depressive disorder (MDD). The data from 27/30 participants (mean age = 30.2 years; SD = 7.9; range = 18–48; 15 females) was included. One participant’s data was excluded due to excessive movement artefact affecting data quality and two due to interruptions in the infusion. Separate data from the participants in the current study have been published previously [15, 16, 20], and full details of the cohort can be found in these publications (a summary and full inclusion and exclusion criteria are provided in the Supplementary Material).

Participants received racemic ketamine on one study visit and the active placebo remifentanil hydrochloride (Ultiva®, GlaxoSmithKline, Auckland, NZ) on the other, the order of which was randomised and counterbalanced with a minimum three-week washout period. Participants provided informed written consent. This study was approved by the Health and Disabilities Ethics Committee, New Zealand (15/NTB/53) and the trial was registered at*:* https://www.anzctr.org.au/Trial/Registration/TrialReview.aspx?id=368052&isReview=true, registration number: ACTRN12615000573550.

### Drug administration

All interventions were administered by an anaesthetist. Drugs were delivered via intravenous cannula in the left antecubital fossa in a magnetic resonance imaging (MRI) environment. Ketamine was administered as a 0.25 mg/kg bolus, followed by a 0.25 mg/kg/h infusion for 45 min. The active placebo remifentanil was administrated as a 9-minute target-controlled infusion to achieve 1.7 ng/mL predicted plasma concentration using the Minto pharmacokinetic model [21, 22]. Where a participant’s body mass index (BMI) exceeded 30 kg/m^2^, they were dosed according to their calculated ideal body weight (IBW). See Sumner, McMillan [15] for a summary of side-effects and blinding.

Antidepressant response for correlation analyses with neuroimaging measures was defined as the percentage change in Montgomery Asberg Depression Rating Scale (MADRS) score at one-day post-ketamine from baseline, and the effect of ketamine vs. active placebo on MADRS score across all time points was assessed using linear mixed modelling with restricted maximum-likelihood estimation.

### EEG data acquisition

EEG data were continuously recorded from 64 channels using standard BrainCap MR caps and BrainAmp MR Plus amplifiers (Brain Products, Munich, Germany). For more information on acquisition parameters see Supplementary Material. EEG resting-state data were collected during a functional MRI (fMRI) resting-state scan in which participants had their eyes open in a supine position, for 7 min pre-ketamine, for two intermediate minutes including and immediately following the ketamine bolus, and for seven minutes during the infusion.

### EEG data analysis

#### Preprocessing

The initial pre-processing steps for the EEG data are presented in the Supplementary Material and have been described previously [20]. The data were epoched into 2200 ms segments, corresponding to volumes collected with fMRI, and then referenced to the common average reference.

#### Spectral analysis

Global covariance matrices were generated, which were obtained after filtering into seven frequency bands using a fourth-order bidirectional Butterworth filter: delta (1–4 Hz), theta (4–8 Hz), alpha (8–13 Hz), low beta (15–26 Hz), high beta (28–40 Hz), low gamma (42–53 Hz), and high gamma (55–67 Hz). These frequencies were chosen to avoid the slice artefact frequency and its harmonics that contaminate EEG data collected simultaneously with fMRI (see data with scanner harmonics in Supplementary Fig. S1). Linearly constrained minimum variance beamforming [23] was applied using 6 mm resolution grids warped to the template head-model provided with FieldTrip [24] to generate spatial filters for each voxel (i.e. 5604 locations) for each frequency band. In order to compute volumetric images of the effects of ketamine on source amplitude, the Hilbert transform was used to obtain the oscillatory amplitude envelope at each location, with the mean amplitude calculated for the pre-infusion and during infusion time periods. Differences at each location were calculated at the group level using paired samples *t*-tests.

The peaks extracted for modelling were selected as in Muthukumaraswamy, Shaw [10]. A Montreal Neurological Institute (MNI) located voxel representing the peak increase in frontal theta near the left medial prefrontal cortex [MNI: −12 36 60] and a voxel representing the peak alpha decrease in the left parietal cortex [MNI: −24 −66 66] were selected from the grand average source localised volume.

### Computational modelling

#### Main study

**Fig. 1:**
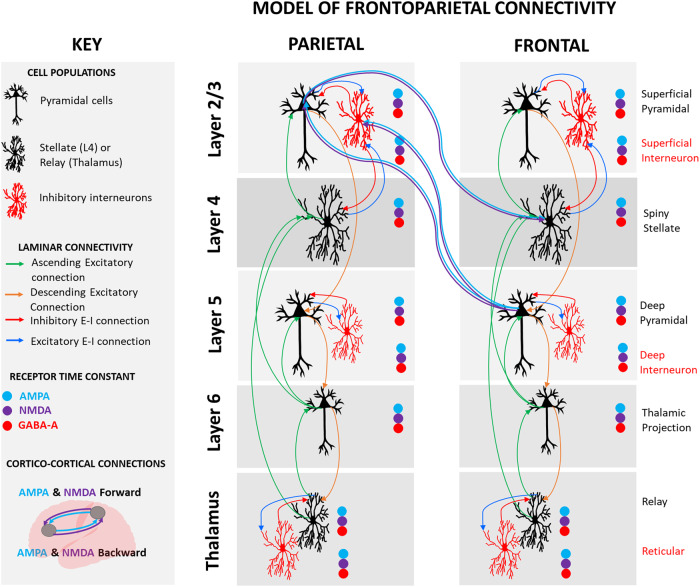
Model architecture and connectivity. Coloured circles indicate that ionotropic AMPA, NMDA and GABA-A receptors were present in each cell population. Excitatory populations are connected by ascending and descending excitatory connections and include superficial pyramidal cells, spiny stellates, deep pyramidal cells, thalamic projections and relay cells. Inhibitory populations are connected by excitatory and inhibitory (E–I) reciprocal connections and include superficial interneurons, deep interneurons, and reticular cells. Cortico-cortical connections are AMPA and NMDA receptor-mediated. Nodes were located in the medial prefrontal cortex [MNI −12 36 60] and superior parietal lobule [MNI −24 −66 66].

The thalamocortical model reported in Shaw, Muthukumaraswamy [18] was fitted to the resting-state EEG data (see Fig. 1 for a schematic of the model). Data were filtered from 3 to 100 Hz and the 2200 ms epochs were averaged according to whether they were recorded in the 7 min before the ketamine infusion, during the 2 min of bolus, and during 5 min of the ketamine infusion immediately following the bolus.

The model itself is an 8-population model that is built upon the earlier 4-population spm_fx_cmm_NMDA.m model available in the SMP12 software (http://www.fil.ion.ucl.ac.uk/spm/) [10, 19]. The extended model additionally contains thalamic relay and deep pyramidal projection populations. Inhibitory interneurons separately act on superficial and deep layers, as well as inhibitory reticular input onto the thalamus. AMPA, NMDA and GABA-A receptors are present in each cell population.

The model equations and parameterisation of the neural and observational model for the intrinsic connections and receptors are outlined in full in ref. [18] and available on GitHub (https://github.com/alexandershaw4/TransPsych_KetDep_GABA_SumnerShaw). From here the model is as in the spm_fx_cmm_NMDA model; extrinsic connections are AMPA and NMDA receptor-mediated as in Muthukumaraswamy et al. [10]. Specifically modelled connections and receptors are depicted in Fig. 1 and are informed by the canonical microcircuit proposed by Douglas and Martin [25] based upon Gilbert and Wiesel [26]. Our choice of model rests upon the observation that cortical-only (e.g. canonical microcircuit) models exhibit a limited repertoire of simultaneous frequency responses, insufficient for reproducing broadband spectral changes induced in our data. Accordingly, we have shown that our extended model overcomes this bandwidth limitation and outperforms canonical cortical models in Bayesian model selection [18] even when correcting for the increased number of parameters.

The model was fitted using standard Dynamic Causal Modelling (DCM) for spectral densities as in Moran et al. [27], which uses free energy as the objective function, such that the objective function is not just a likelihood (i.e. goodness of fit) term but rather an accuracy term minus complexity term. Thus, the reported result represents not just the best fit of the model spectrum to the data, but the ‘simplest best fit’ in terms of parameter updates.

A repeated measures (RM) ANOVA was used to assess the effect of ketamine on parameters pre-infusion, during the bolus and during the infusion. Parameters analysed include extrinsic AMPA and NMDA backward and forward connections between the parietal and frontal nodes. Intrinsic connections analysed included self-gain on superficial pyramidal (SP) cells, superficial inhibitory interneuron (SI) input onto SP, relay (RL) onto spiny stellate (SS) and thalamic pyramidal (TP) cells onto RL in both parietal and frontal nodes. Time constants analysed included frontal and parietal AMPA, GABA-A and NMDA receptor populations separately.

Connectivity parameters were analysed by connection and time constant as separate ANOVAs. Correction for multiple comparisons was applied to the univariate comparison output within each ANOVA run using Benjamini and Hochberg’s false discovery rate (FDR) [28].

Pearson’s correlations were run on the absolute change in MADRS score at 24-h and 7 days post-ketamine and the parameters that were significantly changed by ketamine when uncorrected for multiple comparisons. Correlations were corrected for multiple comparisons as above using FDR.

#### Repeatability of ketamine’s effects

In order to test whether the model would produce the same pharmacodynamic effects of ketamine a second dataset was used that was collected under equivalent conditions. Ketamine was administered as in the section “Drug administration”. The EEG data were collected as in the section “EEG data acquisition” and the EEG pre-processed and prepared for modelling as in the section “Computational modelling”. These data are published in Forsyth, McMillan [29]. This dataset comprises 30 healthy male participants without MDD (mean age = 27.3 years; SD = 6.2; range = 19–37).

## Results

### Clinical mood effects

The significant antidepressant response of these participants has been reported extensively elsewhere [15, 16, 20, 30]. The absolute reduction in MADRS from baseline to 24 h post-ketamine, as used in the correlations, can be found in the Supplementary Material.

### Volumetric ketamine-induced EEG spectral changes

Volumetric ketamine-induced EEG spectral changes were computed in the source space, comparing the pre-ketamine baseline to ketamine infusion data and are presented in the Supplementary Material. Consistent with similar analyses in healthy volunteers [10, 31] and the findings in a subset of these participants [20], ketamine significantly decreased spectral amplitude in the delta, alpha, and low beta bands, while increases in spectral amplitude were observed in the high beta, low gamma, and high gamma frequency bands. The theta frequency band displayed a relatively small increase in frontal spectral amplitude and a posterior decrease.

### Modelling the pharmacodynamic effects of ketamine

Models provided a good fit for all individual spectral densities (>90% variability explained), model fit compared to the data are plotted in the Supplementary Material.

**Fig. 2:**
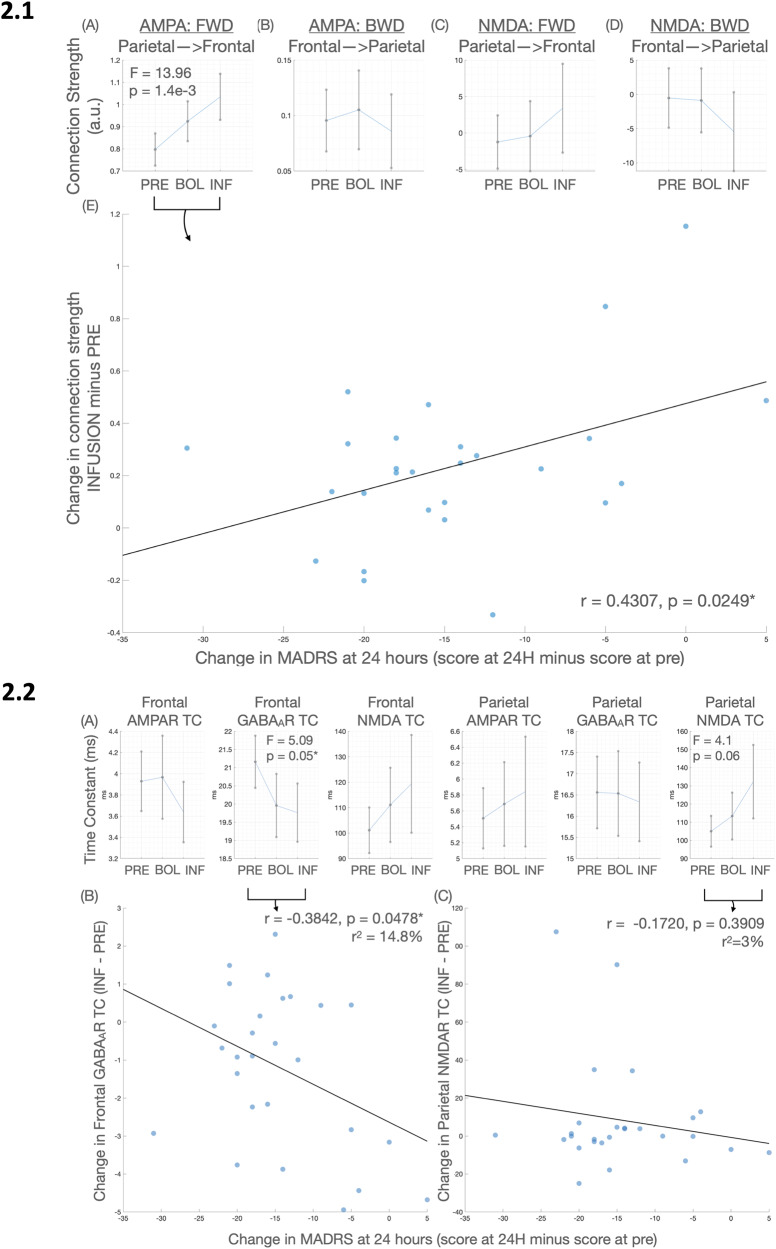
Graphs of parameter output and correlations of changes in parameters with MADRS. (Panel 2.1) **A**–**D** shows the means and standard error of the mean of forward and backward extrinsic connectivity for the pre (PRE), bolus (BOL) and infusion (INF) time points. Only (**A**), forward, AMPA mediated connectivity, was significant in a repeated measures ANOVA. This change (infusion minus pre) was correlated with the change in depressive symptoms at 24 h, as measured by the MADRS (box **E**). (Panel 2.2) **A** shows the means and standard error of the mean of the AMPA, NMDA and GABA_A_ receptor time constants for each region, at each time point (pre, bolus and infusion). Before FDR correction, both the frontal GABA_A_R and parietal NMDA receptor demonstrated significant effects, however, only the frontal GABA_A_R effect (of a reduced time constant), survived correction. This change in GABA_A_R time constant correlated with a change in depressive symptoms at 24 h post-infusion (box **B**, measured as change in MADRS at 24 h vs. pre-infusion). There was no statistically significant correlation for the parietal NMDAR time constant (box **C**).

**Fig. 3:**
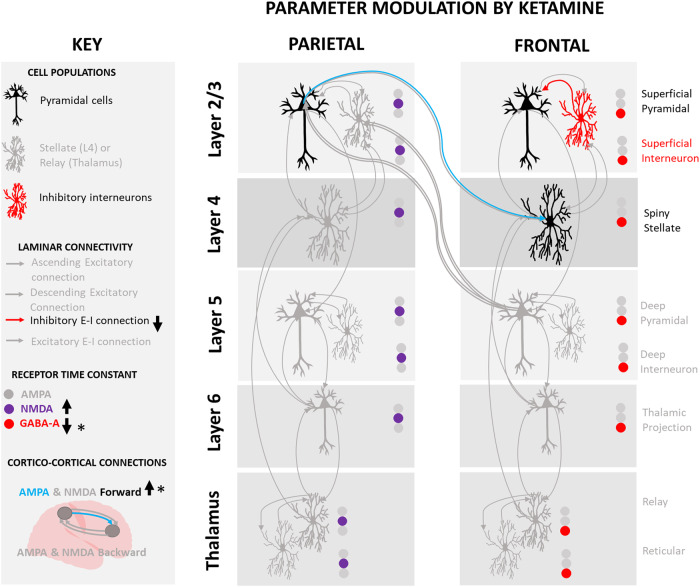
Ketamine significantly increased AMPA forward connectivity (superior parietal to medial prefrontal cortex) and the GABA-A time constant decreased. These results survived FDR correction for multiple comparisons (indicated by * in the key). Inhibitory input from superficial interneurons to superficial pyramidal cells was decreased and the NMDA time constant increased though these results did not survive FDR correction.

A summary of the RM ANOVA results is presented in Table 1. Ketamine significantly increased AMPA forward connectivity (parietal to frontal) in the bolus and infusion in an apparently linear fashion (Fig. 2, Panel 2.1A) but did not alter AMPA backward connectivity or NMDA receptor-mediated connectivity (Fig. 2, Panel 2.1B–D). Figure 3 depicts these results in the thalamocortical model.

**Table 1:** Thalamocortical model parameter changes.

		*F*_(DF)_	*P* uncorr	*P* FDR
Extrinsics	AMPA Forward	13.95_(2,26)_	1.4 × 10^−5^	1.4 × 10^−3^
	AMPA Backward	0.94_(2,26)_	0.39	0.39
	NMDA Forward	0.24_(2,26)_	0.24	0.32
	NMDA Backward	0.16_(2,26)_	0.16	0.31
Intrinsics	Frontal SP to SP	1.73_(2,26)_	0.18	0.22
	Frontal SI to SP	3.37_(2,26)_	0.04	0.16
	Frontal RL to SS	2.35_(2,26)_	0.11	0.18
	Frontal TP to RL	2.35_(2,26)_	0.11	0.18
	Parietal SP to SP	2.87_(2,26)_	0.07	0.16
	Parietal SI to SP	0.56_(2,26)_	0.57	0.57
	Parietal RL to SS	0.59_(2,26)_	0.56	0.57
	Parietal TP to RL	0.59_(2,26)_	0.56	0.57
Time constants	Frontal AMPA	1.86_(2,26)_	0.16	0.24
	Frontal GABA	5.09_(2,26)_	0.009	0.050
	Frontal NMDA	2.52_(2,26)_	0.09	0.18
	Parietal AMPA	0.46_(2,26)_	0.63	0.75
	Parietal GABA	0.12_(2,26)_	0.89	0.89
	Parietal NMDA	4.10_(2,26)_	0.02	0.06

Ketamine significantly decreased the frontal GABA-A population time constant with the majority of this decrease occurring in the bolus (Table 1, Fig. 2, Panel 2.2A). Uncorrected, the parietal NMDA population time constant increased linearly and intrinsic superficial inhibitory intraneuronal input onto frontal superficial pyramidal populations decreased linearly. These two results did not survive FDR correction.

The increase in AMPA-mediated forward connectivity was significantly correlated with a smaller decrease in MADRS 24-h post-ketamine (*r* = 0.43, *p* = 0.02) (Fig. 2, Panel 2.1E). The GABA-A receptor time constant was also significantly correlated with change, smaller decrease in the GABA-A receptor time constant was also related to greater MADRS reduction (*r* = −0.38, *p* = 0.04) (Fig. 2, Panel 2.2B). There was no significant correlation between the NMDA receptor time constant and MADRS change. By 7 days post-infusion, only the decrease in the GABA-A time constant was still significantly correlated with MADRS reduction (see Supplementary Material).

### Repeatability of ketamine’s effects in healthy volunteer dataset

The thalamocortical model used provided a good fit for all individual spectral densities with >90% variability explained.

**Fig. 4:**
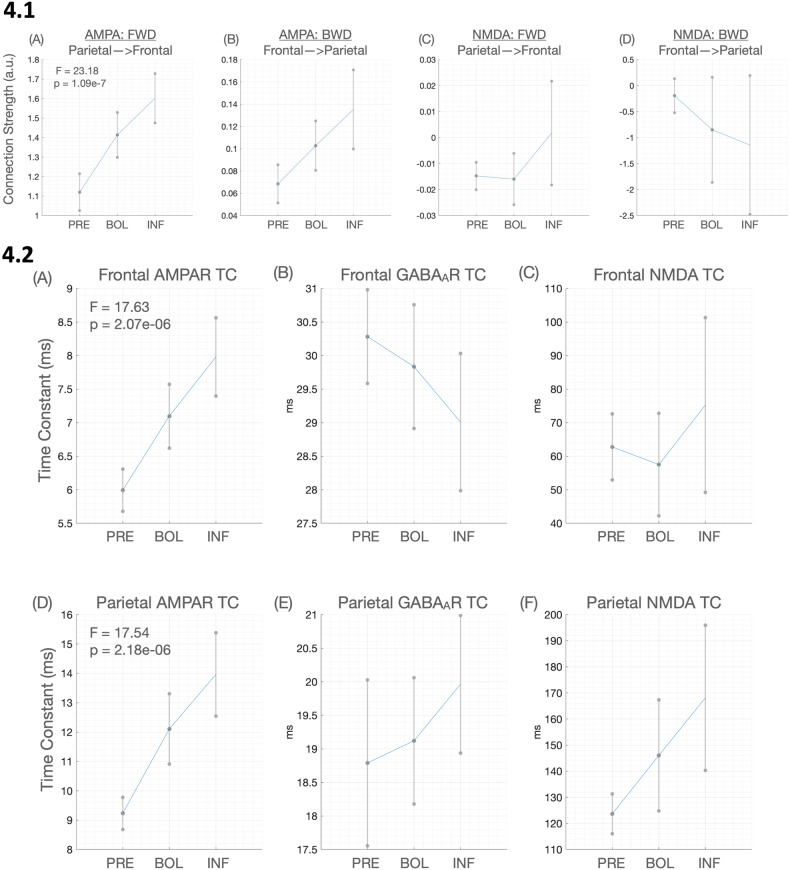
Graphs of parameter output for healthy volunteer dataset. (Panel 4.1) **A**–**D** shows the means and standard error of the mean of forward and backward extrinsic connectivity for the pre, bolus and infusion time points for the second dataset (healthy individuals). As per dataset one, only (**A**), forward, AMPA mediated connectivity, was significant in a repeated measures ANOVA. (Panel 4.2) **A**–**F** shows the means and standard error of the mean of the AMPA, NMDA and GABA-A receptor time constants for each region, at each time point (pre, bolus and infusion) for dataset 2 (healthy individuals). Unlike dataset 1, the change in GABA-A receptor time constant was not significantly altered by ketamine. Rather, significant changes in AMPA receptor time constants (**A** and **D**)—getting slower—were observed in both the frontal and parietal regions.

A summary of the RM ANOVA results is in Table 2. Significantly increased AMPA forward connectivity was consistent with the main dataset (Fig. 4, Panel 4.2A). Decreased superficial interneuronal input onto superficial pyramidal cells was consistent with the original dataset though in this sample the result was highly significant and survived correction. Decreased frontal self-gain on superficial pyramidal cells was also significant. All other intrinsic parameters were also significantly changed by ketamine. Parietal superficial interneuronal input onto superficial pyramidal cells and self-gain on pyramidal cells were significant. Thalamocortical connections were all significantly increased by ketamine including frontal and parietal relay to spiny stellates and thalamic pyramidal to relay parameters.

**Table 2:** Thalamocortical parameter changes in comparison dataset of healthy males without depression.

	Parameter	*F*_(DF)_	*P* uncorr	*P* FDR
Extrinsics	AMPA Forward	23.18_(2,23)_	1.09 × 10^−7^	0.0000
	AMPA Backward	3.15_(2,23)_	0.052	0.10
	NMDA Forward	1.33_(2,23)_	0.27	0.36
	NMDA Backward	0.74_(2,23)_	0.48	0.48
Intrinsics	Frontal SP to SP	12.77_(2,23)_	3.87 × 10^−6^	0.0003
	Frontal SI to SP	10.48_(2,23)_	1.78 × 10^−4^	0.0007
	Frontal RL to SS	4.32_(2,23)_	0.02	0.02
	Frontal TP to RL	4.32_(2,23)_	0.02	0.02
	Parietal SP to SP	4.43_(2,23)_	0.02	0.02
	Parietal SI to SP	3.42_(2,23)_	0.04	0.04
	Parietal RL to SS	4.93_(2,23)_	0.01	0.02
	Parietal TP to RL	4.93_(2,23)_	0.01	0.02
Time constants	Frontal AMPA	17.63_(2,23)_	2.07 × 10^−6^	0.000
	Frontal GABA	0.80_(2,23)_	0.46	0.48
	Frontal NMDA	0.74_(2,23)_	0.48	0.48
	Parietal AMPA	17.54_(2,23)_	2.18 × 10^−6^	0.000
	Parietal GABA	0.75_(2,23)_	0.48	0.48
	Parietal NMDA	3.35_(2,23)_	0.04	0.09

Summarised in Table 2, interestingly the GABA receptor time constant was not significantly changed. Instead, the AMPA receptor time constant in the parietal and frontal node was significantly increased (Fig. 4, Panel 4.2A, D). Parietal NMDA receptor time constant also increased (Fig. 4, Panel 4.2F) though did not survive FDR correction.

## Discussion

The current study investigated resting-state EEG before an infusion of ketamine for depression, during the bolus and during the infusion. During the infusion, ketamine significantly modulated spectral amplitude in all seven frequency bands analysed and was largely typical of those documented in healthy volunteers [10, 11, 31–33].

A thalamocortical model of laminar microcircuitry in the medial prefrontal cortex and superior parietal lobe was fitted to the spectra using DCM, based on previous work on ketamine’s modulatory effects on the brain [10–13] (Fig. 1). The study revealed both ketamine bolus and infusion mediated increases in AMPA frontoparietal forward connectivity (from parietal to frontal) and decreases in the frontal GABA-A receptor time constant (Fig. 3). Increased parietal NMDA receptor time constant and decreases in the inhibitory intraneuronal input into superficial pyramidal cells did not survive multiple comparisons correction (Fig. 3). Together these results are consistent with the known effects of ketamine from pre-clinical work (as reviewed in refs. [5, 34]). The significant changes to AMPA frontoparietal connectivity and GABA-A receptor time constant were significantly correlated with the antidepressant response to ketamine at 24-h. The correlation with the GABA-A receptor time constant was still significant at 7 days post-ketamine.

A comparison dataset was used to look at consistency in model output with ketamine. The dataset used had been collected and pre-processed under equivalent conditions. The key difference was that the cohort was healthy young males. Many of the parameter changes that the model output produced were in the same direction as the cohort with MDD—however in general more parameters were statistically significant in this dataset than in the cohort with MDD including increased thalamocortical connectivity. The reduced number of significant effects in the depressed dataset may be driven by the greater heterogeneity of this cohort including factors such as sex, age, concomitant medications, comorbidities as well as depression. This heterogeneity may have both reduced power to detect significant changes or cause inherent physiological differences in the response to ketamine between the cohorts.

### Contextualising the finding with data previously analysed from the main cohort with MDD

In the current results, the more the GABA-A receptor time constant was reduced and the more AMPA frontoparietal connectivity was increased by ketamine the less likely a person was to experience an antidepressant response (Fig. 2, Panels 2.1E and 2.2B). The concept of ketamine causing a greater effect in the brain for those who go on to have less antidepressant response appears paradoxical. Previous publications report that in this same dataset a more intense experience; specifically experiences of unity, spirituality, and insight measured using the 11D-ASC, were correlated with a greater antidepressant response [30] indicating that these participants who did not respond were having a subjective less intense experience. 3-h post-ketamine, a greater increase in bottom-up connectivity in response to unexpected sensory input (auditory mismatch response) was related to greater antidepressant response indicating an improvement in sensitivity to prediction errors and short-term plasticity mechanisms [16]. This suggests downstream, responders would have greater plasticity improvements than non-responders.

However, there are also previous results from this dataset that are consistent with this finding of greater acute changes being related to lower antidepressant response. Blood oxygen level-dependent (BOLD) signal was significantly increased in the participants with lower MADRS improvements in the simultaneously recorded resting state fMRI [20]. This occurred in many spatially distributed regions across the brain. Therefore, while initially appearing paradoxical, the result sits within the wider context of data from this cohort.

### AMPA-mediated forward connectivity correlation with antidepressant response

Within the wider literature, a greater change in AMPA forward connectivity has previously been related to the antidepressant response. Specifically, in depression, 6–9 h post-ketamine during a tactile task and using a four-population model (cmm_NMDA), larger changes in AMPA forward connectivity from somatosensory (parietal) to frontal cortex were shown to be correlated to change in MADRS [14]. This suggests our finding of acute changes may have endured until after the infusion, and that changes to forward AMPA-mediated connectivity are emerging as a reliable correlate of antidepressant response to ketamine. Further, the current study adds to growing evidence that ketamine consistently changes frontal and frontoparietal connections and this effect is robust to a range of effective and functional connectivity analysis techniques [10–13].

### GABA-A receptor time constant correlation with antidepressant response

The role of GABAergic inhibitory interneuronal neurotransmission in medial prefrontal cortex and depression is established [35] and has widely been described as important to the antidepressant response to ketamine [34]. Disinhibition of GABA-A-mediated interneurons is most specifically captured in two parameters of the model: the superficial inhibitory interneuron input into superficial pyramidal cells and the GABA-A receptor time constant. Reduction in inhibitory interneuron input into superficial pyramidal cells has previously been shown with a four-population model (spm_fx_cmc.m) following a ketamine infusion [36]. In the current study, both superficial inhibitory interneuron input into superficial pyramidal cells and the GABA-A receptor time constant were decreased by the ketamine bolus and infusion, though only the GABA receptor time constant survived correction. Interestingly while the comparison dataset of healthy young men tended to show greater significant changes in the same parameters as well as additionally significant parameters during the infusion, the decrease to the GABA time constant was not significant.

The addition of GABA-A receptors is unique to the current model and their change under ketamine was found to be related to the magnitude of the antidepressant response. In the type of modelling employed for this study the time constants can be considered a lumped parameter of receptor kinetics, however, changes detectable in mesoscale (i.e. spectra from a local field potential) are likely to contain information including changes to numerosity and density of a receptor, (a)synchronous opening, as well as the channel opening time and decay constant itself. GABA-A receptor dysfunction is well documented in depression, with deficits in GABA found [37]. Increased GABA receptor mRNA expression in people who die by suicide may be compensatory to low levels of GABA [35, 38].

We speculate that the greater reactivity of the GABA-A receptor time constant in people who responded less to ketamine points to a larger GABA dysfunction. For example, because ketamine increases the sensitivity of GABA-A receptors to low concentrations of GABA [39], participants with lower GABA [37] might have experienced the greatest change from their baseline.

### Strengths and limitations

One of the strengths of this study was the availability of a control group to compare to the main cohort with MDD. However, the ability to compare the groups was limited by them being non-age, medication or sex-matched. For this reason, we chose not to undertake any quantitative comparisons of the two cohorts. Overall, the similarities in synaptic parameter changes between the groups speak to the reliability of parameter recovery and drug effects, this includes the direction of changes even when not significant (Fig. 2, Panel 2.1A–D compared to Fig. 4, Panel 4.1A–D and Fig. 2, Panel 2.2A compared to Fig. 4, Panel 4.2A–F). When considering these similarities it is important to note that unlike many spectral DCM studies (for example [10]), we did not use a general linear model contrast to encourage a given direction of change.

Computational models of generated electrophysiology data are limited in that they do not provide an estimate of every potential parameter in the brain (i.e. every channel and every cell type). However, they do represent a mesoscopic and biologically plausible representation of the major contributors to electrophysiologically generated signal, and in the current model and similar models fitted with DCM, parameter estimates are constrained to biologically informed priors and expected posterior estimation distributions. One of the greatest sources of evidence that the models are complementary to invasive electrophysiology and pharmacodynamic investigations is when the models produce changes that are plausible and realistic. The current data and TCM output provide a strong example of this.

### Future directions and conclusion

The current data suggest that the model used, with further validation and testing may have a future role in treatment augmentation. For example, people with smaller MADRS changes and larger GABA receptor time constant reductions and AMPA connectivity increases might benefit from different infusion schemes, for example, a lower and slower drug administration to regulate the cortical response. Alternatively, these data may be indicative of a marker of people who will not respond to ketamine, in which case the model could be used to prevent unnecessary further ketamine doses and expedite movement to another potentially more beneficial treatment. Such hypotheses could be tested and confirmed using the methods and model used in the current study.

Overall this study supports the large body of literature suggesting the acute mechanism underlying ketamine’s antidepressant effects are related to GABA-A and AMPA receptor properties rather than NMDA receptor antagonism. While generative computational modelling is limited to only describe the data in terms of their parameters if further validated, the relationship between key GABA and AMPA receptor-mediated parameters indicates modelling also could be used as a precision medicine tool during a patient’s first infusion, informing their individual regimen including dose, infusion rate (if relevant) and augmentative and complementary therapies. Such validation could include modelling pre-clinical work, or further drug studies in humans that perturb the GABA, AMPA and other systems.

### Supplementary information

Supplement: Neurophysiological evidence that frontoparietal connectivity and GABA-A receptor changes underpin the antidepressant response to ketamine

## Data Availability

The data underlying this article cannot be shared publicly as it was not part of the original ethics application to do so. The data will be shared on reasonable request to the corresponding author.
