# Supplementary material for: Neurophysiological evidence that frontoparietal connectivity and GABA-A receptor changes underpin the antidepressant response to ketamine
Source: Transl Psychiatry. 2024 Feb 24;14:116. doi: 10.1038/s41398-024-02738-w (PMC10894245; doi:10.1038/s41398-024-02738-w)
Supplement: Supplementary file 1 — Supplement: Neurophysiological evidence that frontoparietal connectivity and GABA-A receptor changes underpin the antidepressant response to ketamine [file 41398_2024_2738_MOESM1_ESM.docx]

**Neurophysiological evidence that frontoparietal connectivity and GABA-A receptor changes underpin the antidepressant response to ketamine**

***Supplementary Material***

**Methods**

Full inclusion and exclusion criteria are reproduced with permission from (1) in Table S1 and Table S2.

**Table S1. Outline of the ketamine and depression clinical trial inclusion criteria.**

| **Inclusion criteria** | | |
| --- | --- | --- |
| **Consent** |  | Willing and able to give informed consent for participation in the trial |
| **Demographics** | Age | 18 – 60 years |
|  | Sex | Male or female |
| **Mental health** | Diagnosis | Major depressive disorder according to DSM-V criteria |
|  | Duration | Greater than 3 months |
|  | MADRS | >20 (i.e. moderate to severe depression) |
|  | Treatment status | Inadequate response to at least two antidepressant courses in the lifetime of their depression |
|  |  | Stable on current medication for at least four or more weeks prior to Study Day 1 |

**Table S2. Outline of the ketamine and depression clinical trial exclusion criteria.**

| **Exclusion criteria** | | |
| --- | --- | --- |
| **Consent and communication** |  | Inability to speak or read English |
| **Mental health** | Lifetime | History of psychosis |
|  |  | History of abuse of ketamine or phencyclidine |
|  | Current | Any unstable medical or neurologic condition, judged at the discretion of the clinician |
|  |  | Imminent risk of suicide as determined by the MADRS/clinical interview |
|  |  | Substance abuse or dependence in previous 6 months |
|  | Treatment status | Planned major changes to psychotropic medication |
|  |  | Planned or probable use of ECT |
| **Drug contraindications** |  | Significant renal or hepatic impairment |
|  |  | Cardiovascular conditions including abnormal heart rate and blood pressure checked at screening |
|  |  | Female participant who is pregnant, lactating or planning pregnancy during the course of the trial |
|  |  | Planned use of ketamine, for example, for pain control |
|  |  | Current use of NMDA antagonist medications (e.g. memantine, amantadine, rimantadine, dextromethorphan, or procyclidine) |
|  |  | Contraindication to the use of ketamine/remifentanil according to manufacturer guidelines |
|  |  | Regular use of any medication deemed to be contraindicating as judged by the attending study physicians |
| **Other safety criteria** |  | Participants who have participated in another research trial involving an investigational product in the past 12 weeks |
|  |  | Body weight <50kg or >120kg |
|  |  | Inability to fast for two hours prior to each administration of trial drug |
|  |  | Any other condition judged by the treating clinician as likely to impact on the ability of the participant to complete the trial |
| **MRI** |  | Contraindications for MRI scanning |

**Table S3. Summary of cohort demographics.**

| **Age** (mean, std. dev) | 30.2 | | 8.2 |
| --- | --- | --- | --- |
| **Sex** (n F, %) | 15 | | 50 |
| **MADRS Score at screening** (mean, std. dev) | 29.5 | | 5.7 |
| **Number of failed treatments**1 (median, range) | 3 | | 2-8 |
| **Comorbid anxiety** (n, %) |  | 20 | 76.9 |
| **Length of illness2** (n, %) | < 5 years | 6 | 23.1 |
|  | 5 – 10 years | 7 | 26.9 |
|  | > 10 years | 11 | 42.3 |
| **Current treatment3** (n, %) | SSRI | 9 | 34.6 |
|  | SNRI | 5 | 19.2 |
|  | Tricyclic | 1 | 3.8 |
|  | Other antidepressant | 3 | 11.5 |
|  | Augmentation | 1 | 3.8 |
|  | Counselling | 2 | 7.7 |
|  | Nil | 10 | 38.5 |
| **Months on current treatment** (median, range)4 |  | 8.75 | 1.5-24 |

1 Lifetime, not only current episode. Underestimates the actual number of failed treatments as only includes those that could be specifically recalled by the participant. Includes counselling.

2 Approximate and based on self-report. Lifespan of depression, not only current episode. Two participants did not report the length of their illness.

3 Five participants were receiving a combination of treatments: SSRI and other antidepressant (1), SNRI and other antidepressant (1), SSRI and counselling (1), SNRI and counselling (1), and other antidepressant and augmentative treatment 1).

4 At a stable dose

The following power calculations were performed in G*Power 3.1 (1). With a sample size of 30, a significance-level of α = 0.05, β = 0.8 we are powered to see effect sizes of 0.6 which is classified as a small to medium effect size.

*EEG acquisition.* EEG data were continuously recorded from 64 channels using the Standard BrainCap MR and BrainAmp MR Plus amplifiers (Brain Products, Munich, Germany). The ground electrode was located at AFz, reference electrode at FCz, and a drop-down electrode attached centrally to the participant’s back for recording of electrocardiography (ECG). EEG data were recorded with BrainVision Recorder software (Brain Products GmbH, Munich, Germany) and sampled at 5 kHz with a resolution of 0.5 μV, range of ± 16.384 μV, and a bandwidth of 0.1 to 250 Hz. Impedances were typically kept below 10 kΩ.

A Syncbox device (Brain Products GmbH, Munich, Germany) was used to synchronise the EEG recording to the MR scanner clock. The amplifier system was positioned on a sled behind the participant within the scanner bore to reduce cable length. The cables connecting the cap and the amplifiers were fixed with adhesive tape to prevent any additional movement-related artefacts.

*Preprocessing.* The scanner-induced gradient artefact, caused by the fast switching of the MR gradients during fMRI acquisition, was reduced by realignment parameter-informed template subtraction (2), a modified version of standard template artefact subtraction (3). The data were subsequently downsampled to 500 Hz and bandpass filtered to between 0 and 100 Hz. The ballistocardiogram (BCG) artefact, caused by the pulsatile motion of blood in the head, was corrected using an automated approach combining ICA with singular value decomposition (SVD) to remove and/or filter components from the data which share high levels of mutual information with the cardiac trace (4).

*Spectral analysis***.**


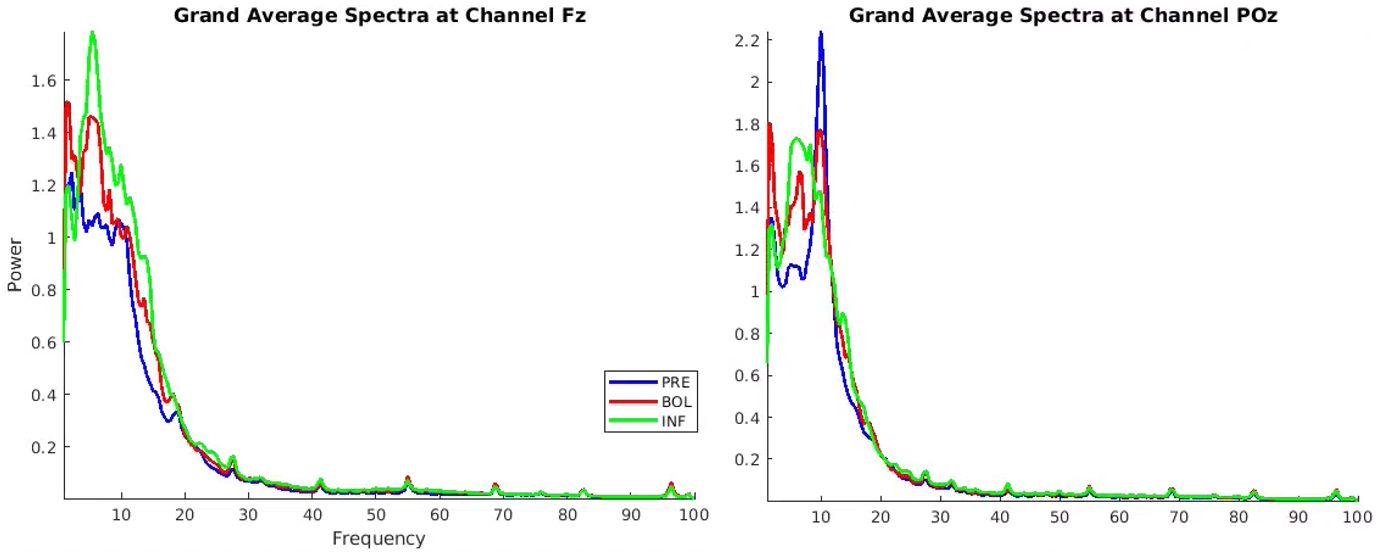


*Figure S1. Resting-spectra collecting pre-infusion, during the bolus and infusion. The scanner harmonic is evident as small peaks at regular intervals that are not different between recordings. To avoid these in the spectral analysis however we cut the bands as follows: delta (1-4 Hz), theta (4-8 Hz), alpha (8-13 Hz), low beta (15-26 Hz), high beta (28-40 Hz), low gamma (42-53 Hz), and high gamma (55-67 Hz).*

**Results**

**Table S4. MADRS results**

| **Participant**  **number** | **24-hours**  **Post-ketamine minus Baseline** | **7-days**  **Post-ketamine minus Baseline** |
| --- | --- | --- |
| **1** | -20 | -14 |
| **2** | -21 | -19 |
| **3** | -14 | -8 |
| **4** | 0 | 0 |
| **5** | -22 | -20 |
| **6** | -6 | -5 |
| **7** | -17 | -10 |
| **8** | -13 | -18 |
| **9** | -5 | -5 |
| **10** | -9 | -5 |
| **11** | -5 | -1 |
| **12** | -18 | -20 |
| **13** | -16 | -16 |
| **14** | -20 | -15 |
| **15** | -18 | -18 |
| **16** | -23 | -20 |
| **17** | 5 | 4 |
| **18** | -15 | 0 |
| **19** | -14 | -2 |
| **20** | -4 | 0 |
| **21** | -18 | -4 |
| **22** | -21 | -17 |
| **23** | -15 | -10 |
| **24** | -16 | -14 |
| **25** | -20 | -15 |
| **26** | -12 | -4 |
| **27** | -31 | -22 |

*Source based EEG analysis*

Volumetric ketamine-induced EEG spectral changes were computed in the source space, comparing pre-ketamine baseline to post-ketamine data. Consistent with similar analyses in healthy volunteers (5) and the findings in a subset of these participants (6), ketamine significantly decreased spectral amplitude in the delta, alpha, and low beta bands, while increases in spectral amplitude were observed in the high beta, low gamma, and high gamma frequency bands (Figure S1). The theta frequency band displayed a relatively small increase in frontal spectral amplitude and a posterior decrease. This differs from analyses in which the ketamine bolus period is included; the bolus delivery induces a large transient increase in the frontal theta spectral power that is not seen here.


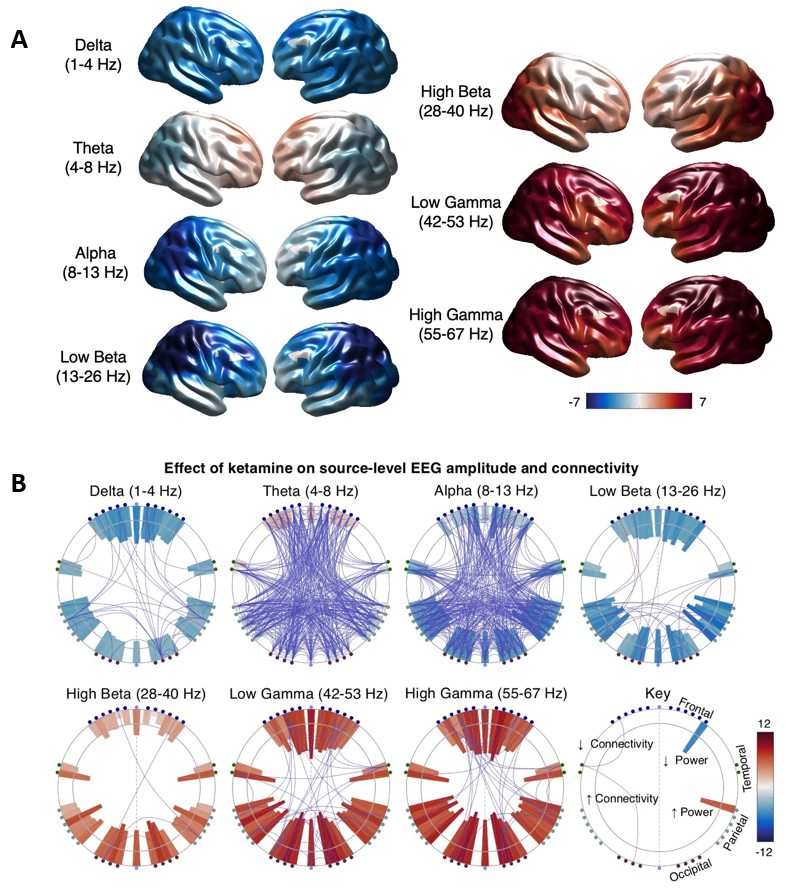


Figure S2. A) Unthresholded t-statistic images of changes in volumetric spectral amplitude in seven frequency bands that span from 1 to 67 Hz for representation of the spatial distribution of ketamine-induced spectral amplitude changes. Contrasts of spectral amplitude represent the difference of ketamine after-and-before infusion. Warm colours represent increases in source power and cool colours represent decreases. The brain surfaces were rendered using the SourceMesh toolbox (https://github.com/alexandershaw4/SourceMesh).

*Model fits to auto- and cross-spectral densities.*


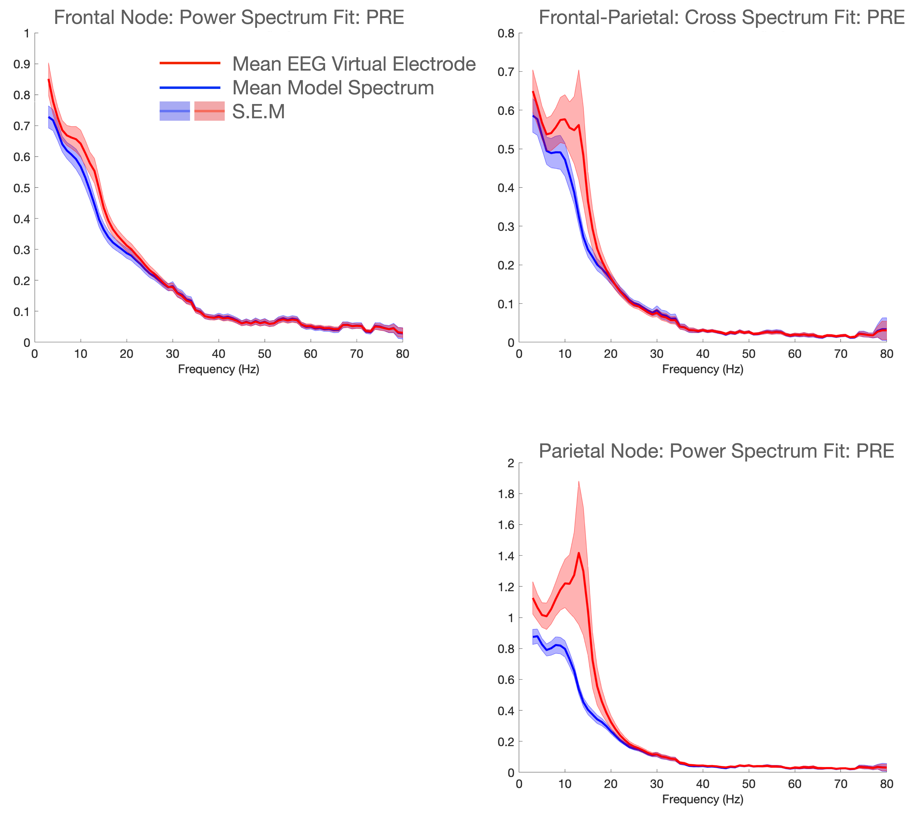


*Figure S3a. Model fits to the frontal and parietal spectra for the pre-ketamine condition.*


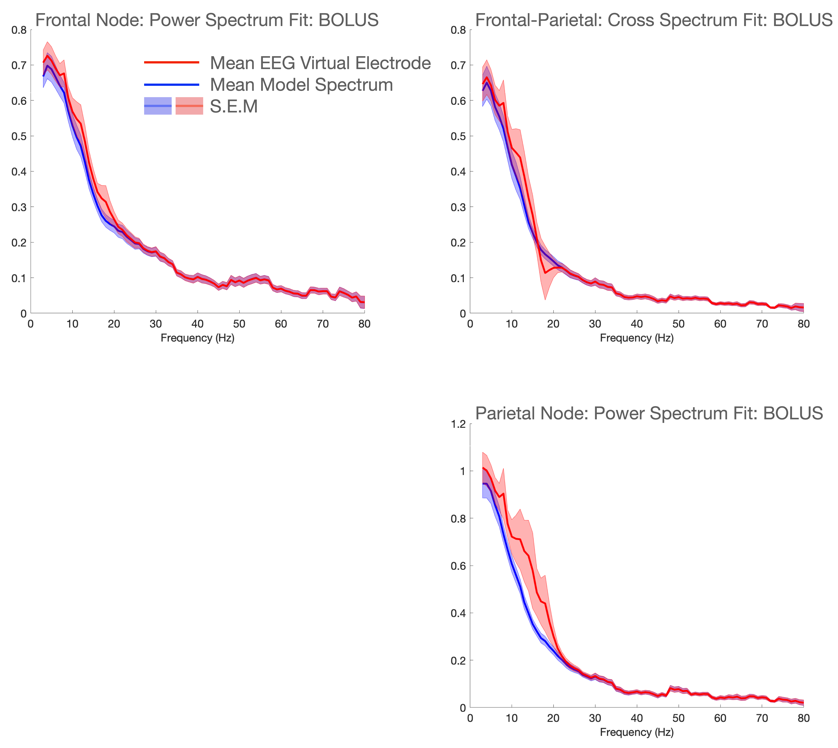


*Figure S3b. Model fits to the frontal and parietal spectra for the bolus-ketamine condition.*


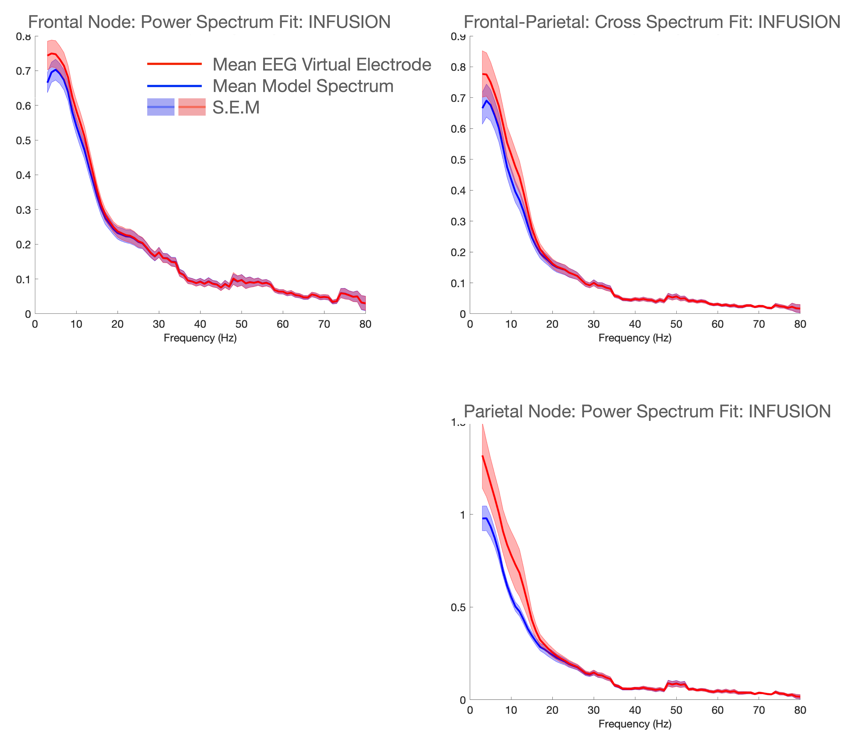


*Figure S3c. Model fits to the frontal and parietal spectra for the infusion-ketamine condition.*

*Parameter correlations with MADRS at 7 days.*

Change in time constants of GABA_A_ receptors in frontal region correlate with change in symptoms 7 days after infusion (MADRS) (Figure S3; Table S5). AMPA backward connections and parietal NMDA receptor time constants were not significantly correlated with MADRS at 7-days (Table S5).

**Table S5.** **Parameter correlations with MADRS at 7 days.**

| Parameter Name | Correlation coefficient | p-value |
| --- | --- | --- |
| Extrinsic Connections | | |
| AMPA (BOL) BKW | 0.2323 | 0.223 |
| AMPA (DUR) BKW | 0.2202 | 0.270 |
| Receptor time constants: frontal | | |
| GABA_A_ (BOL-PRE) | -0.4352 | 0.023* |
| GABA_A_ (DUR-PRE) | -0.4726 | 0.013* |
| Receptor time constants: parietal | | |
| NMDA (BOL-PRE) | -0.1349 | 0.503 |
| NMDA (DUR-PRE) | -0.2309 | 0.247 |

**
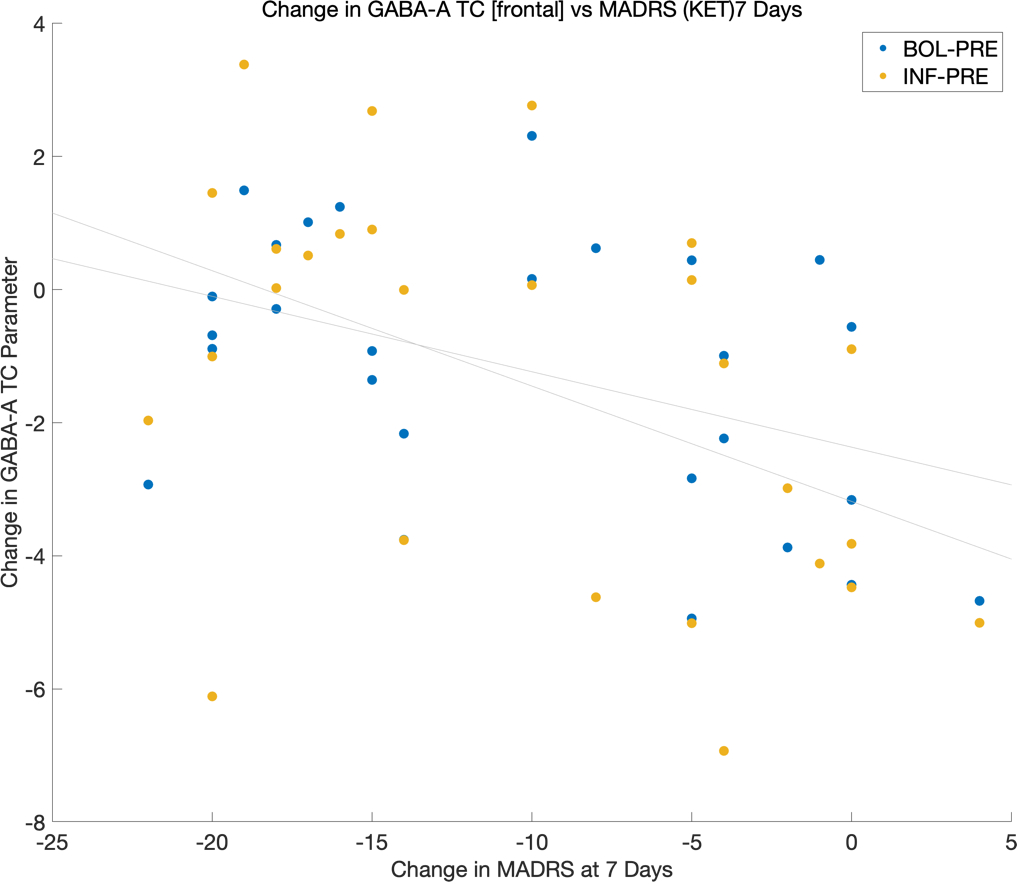
**

*Figure S4. Change in GABA_A_ parameter strength during the infusion compared to baseline correlates with change in MADRS score at 7 days post infusion (r = -0.4726) as well as 24-hours post infusion (r = -0.3842).*

**References**

1. Faul F, Erdfelder E, Lang AG, Buchner A. G*Power 3: a flexible statistical power analysis program for the social, behavioral, and biomedical sciences. Behavior research methods. 2007;39(2):175-91.

2. Moosmann M, Schönfelder VH, Specht K, Scheeringa R, Nordby H, Hugdahl K. Realignment parameter-informed artefact correction for simultaneous EEG–fMRI recordings. NeuroImage. 2009;45(4):1144-50.

3. Allen PJ, Josephs O, Turner R. A method for removing imaging artifact from continuous EEG recorded during functional MRI. NeuroImage. 2000;12(2):230-9.

4. Liu Z, de Zwart JA, van Gelderen P, Kuo L-W, Duyn JH. Statistical feature extraction for artifact removal from concurrent fMRI-EEG recordings. NeuroImage. 2012;59(3):2073-87.

5. McMillan R, Forsyth A, Campbell D, Malpas G, Maxwell E, Dukart J, et al. Temporal dynamics of the pharmacological MRI response to subanaesthetic ketamine in healthy volunteers: A simultaneous EEG/fMRI study. Journal of Psychopharmacology. 2019;33(2):219-29.

6. McMillan R, Sumner RL, Forsyth A, Campbell D, Malpas G, Maxwell E, et al. Simultaneous EEG/fMRI recorded during ketamine infusion in patients with major depressive disorder. Progress in Neuro-Psychopharmacology and Biological Psychiatry. 2020;99:109838.
